# Man's Lost Incisors

**Published:** 1887-02

**Authors:** Bertram C. A. Windle, John Humphreys

**Affiliations:** Dublin


ARTICLE V.

### MAN'S LOST INCISORS.

BY BERTRAM C. A. WINDLE, M. A., M. D., DUBLIN,

AND

JOHN HUMPHREYS, L. D. S. I.

[Read before the British Association and the Central Branch of the British Dental Association.]

Teeth beyond the ordinary number may occur in the incisor portion of the dental series, and fall, when present, into two categories—*supplemental*, that is, incisiform, though generally smaller than the normal incisors, and *supernumerary* or conical teeth, which do not conform to the shape of any other member of the dental series in man. These teeth have been described by various authors, the following being a resume of the literature of the subject. Eustachius states that he has observed two cases of supernumerary teeth, and cites Alexander de Benedictis as having also seen such. Meckel observes that they are more common in the front and in the superior maxilla, and that they differ from the normal teeth in size and shape, being smaller and conical. Generally only a few, as many as eight, have been noticed in each maxilla. This was probably a co-existence of both dentitions. Hunter believes these teeth to be always incisors and canines, whilst Owen goes so far as to state that he has never seen a supernumerary premolar or molar. Wedl gives a lengthy account of both classes of superfluous incisors. The co-existence of six teeth, two being supplemental, in either jaw he considers to be rare—one extra supplemental tooth being more common. Turner has described a case of a slender, conical, supernumerary tooth, situated in the interval between the two upper central permanent incisors. Supernumerary teeth are placed either amongst the permanent teeth or behind them and within the alveolar arch. Their eruption takes place during the first or second dentition, or in the interval between the two; generally, however, they belong to the permanent series.

Baume's interesting remarks upon the subject may be summed up as follows:—The incisive region having formerly contained more than the present number of teeth, it is not strange to find superfluous teeth in various parts of the os incisivum, as the enamel organ, ordinarily abortive, may assert itself and revert to the primitive type. When this occurs, the teeth seldom reach the highest degree of development, as shown by their conical shape. Granting the loss of incisors, in which part of the series does the deficit occur? The Apes have lost a tooth in each incisive bone since the Tertiary period. These animals possess a diastema behind the lateral incisor, which at first sight might appear to point to this spot as the site of the missing tooth. This theory, however, falls to the ground from the fact that animals possessing the full number of six incisors have also a diastema in this position to allow room for the large canine. Baume believes that the medium incisors are those which fail to appear. Amongst the Prosimii and the Cheiroptera, he goes on to say, there is normally a diastema in this position, and this may also be found sometimes in Apes and even in man, though in the latter the width never appears to exceed 2 mm. This separation is more common in the upper than in the lower jaw. Here may sometimes be found superfluous teeth, and here once Baume noticed two excessively small conical teeth co-existing with those normally found.

Dr. Edwards, of Madrid, in a paper entitled "The Missing Incisors in Man: Which are they?" arrives at a similar conclusion to Baume, and on somewhat similar grounds.

On the other hand, Professor Turner, in a paper on "The Relation of the Alveolar Form of the Cleft Palate to the Incisor Teeth and the Intermaxillary Bones." adopts the theory that the missing incisor is the second, In2. His remarks on this vexed question are of such importance that we reproduce them at some length :—

"Not only," he says, "is six a very common number of upper incisors in various mammals, but as is well known to dental surgeons, three incisor teeth are sometimes developed on each side of the human upper jaw when there is no alveolar cleft. I have now before me the casts of two otherwise normal palates taken from different persons by Mr. Andrew Wilson, L. D. S., one with the milk, the other with the permanent dentition, in each of which six upper incisor teeth have been developed. The question therefore naturally arises which of these teeth is suppressed in the normal incisor dentition in man? Some light is thrown on this question by these cases of alveolar cleft. In the cases of double cleft with two incisors in each half of the projecting intermaxillaries, these teeth would be in dental rotation In1, In2, whilst the precanine would be In3, but in many cases of alveolar cleft, more especially when it is one-sided, only one incisor tooth exists between the mesial suture and the cleft, whilst a precanine is present on its outer side. The precanine, as in the preceding example, would be In3, whilst the incisor situated mesially to the cleft would be in the majority if not all of the cases without doubt In1; the suppressed incisor, therefore, would be In2, and it is not unlikely that in normal human dentition the incisor which does not develop is also In3. This view of the homology of the precanine tooth and of the normal lateral incisor, viz., that it is In3, is also advocated by Dr. Allbrecht. Dr. Th. Kolliker, however, has not apparently formed any exact conception of its homology; for although sometimes he refers to it as In3, at others he speaks of it as if it represented the ordinary lateral incisor, which incisor he obviously regards as a different tooth from In3."

This view as to the position of the suppressed tooth is also shared by Mr. Andrew Wilson.

Before proceeding to describe our cases and to make any remarks thereon, we desire to state that we lay but little claim to originality in the theories which we put forward. On the other hand, we believe that the questions which we shall pass in review deserve a more systematic examination than has yet been accorded to them, and that the specimens which we have to describe will in some instances afford additional confirmation, in others throw a new light on the actual facts.

Our specimens have been for the most part obtained by ourselves from the clinique at the Birmingham Dental Hospital, the large number of patients attending at which affords an excellent field for the observation of dental abnormalities.

We have also to express our acknowledgments to the following gentlemen for kindly supplying us with specimens from their collections: Dr. Crapper, of Hanley; Messrs. Chas. Sims and Adams Parker, of Birmingham; Mr. Percy May, of London; and Mr. J. S. Amoore, of Edinburgh.

We have arranged the teeth into eight series:—

Series I. *Supplemental Teeth.*—In this group we have one case of six separate incisors and one of six, the two central being geminous. In both these cases the teeth are in regular series. We have seven in which there were five incisors, in three the additional tooth was on the right side, in four on the left. In all the cases save one they were situated behind the true lateral, generally occasioning some displacement. In one case, however, the intruder was placed between the lateral and central; one case only belonged to the milk dentition. and all were found in the upper jaw.

Series II. *Supernumerary Teeth.*—We have four casts in which there are two of these teeth. In two of these the teeth were situated behind the median incisors; in another one was posterior to the left lateral and a second between the right median and lateral. In the fourth one was posterior to the right central, and the second lay between the two median.

We have fifteen cases in which there was one supernumerary; these teeth were situated inside the alveolar arch posterior to the left median incisor in seven cases, the right in five, and in the middle line in three instances. They generally caused more or less displacement of the remaining teeth. All were found in the superior maxilla and all belonged to the permanent series.

Series III. *Co-existence of supplemental and supernumerary with the normal number of incisors.*—Of this we have one specimen. In it a properly-formed though small incisor is placed behind the right lateral and in series with it, and a blunt tooth posterior to the left median which it displaces forwards. This occurred in the superior maxilla, and belonged to the permanent series.

Series IV. *Substitution of a supernumerary tooth for a normal incisor, the number remaining four.*—Of this we have four specimens. The substitution was one each for the right and left median, and twice for the left lateral. These cases belonged to the superior maxilla and the permanent dentition.

Series V. *Substitution of two supernumerary teeth for normal incisors, the number of teeth remaining four.*—Of this we have six cases, the two lateral incisors being invariably those to suffer.

Series VI. *Absence of one incisor, the number being three.*—In two cases the right lateral (superior) incisor was wanting, and in one the same tooth in the inferior maxilla. All these cases belonged to the permanent series.

Series VII. *Absence of one incisor, diminution or malformation of another, the number being three.*—Of this we have four cases. In two the right lateral was absent, and of these in one case the left was conical and in another small but incisiform. In two the left lateral was absent; in one of these the right was small and incisiform, in the other it was conical. All these belonged to the superior maxilla and permanent dentition.

Series VIII. *Absence of two incisors, the number being reduced to two.*—Of this we have eight cases, of which seven belong to the superior maxilla and all to the permanent series. In all, the laterals are the missing teeth. The jaws are generally well formed, and there are often gaps between the teeth in the incisor region. The ages of four of these patients were respectively 21, 22, 22, 17; of the others we have no exact information, save that they were adults. The eighth case is especially interesting, firstly, as belonging to the inferior maxilla—a rare occurrence, and secondly, as it was found in the same individual as one of the cases in Series VII. In this individual the superior maxilla contained three incisors, one being conical, the inferior only two.

We shall now proceed to discuss in order the various points which a consideration of these series raises:—

1. *Man's original dentition included six incisors.*

The consensus of opinion that man formerly possessed six incisors is so general that we need not linger over the various reasons drawn from comparative anatomy upon which the theory is based, Our desire is to add a further proof from our own specimens.

If, as is now generally conceded, supplemental and supernumerary teeth are to be considered as reversions to the original type, then we have in one of our cases, in so far as the upper jaw is concerned, an instance of the complete archaic human incisor dentition. Instances of this kind are, as Wedl observes, very rare. Galton quotes a case in which two perfectly formed supplemental lateral incisors were placed behind the normal lateral incisors in the upper jaw. Our case differs from this in the fact that in it the incisors are in perfect series. Mr. A. Wilson mentions three cases of this nature, one of which occured in the temporary dentition. Professor Flower records a case observed in the skull of an adult Englishman in the museum of the Royal College of Surgeons of England, in which there were six incisors in the upper jaw, the supplemental pair being placed behind the normal incisors. Dr. Edwards figures a case in which there were five incisors, one of the median being geminous; this he regards as an instance of the co-existence of six incisors. We have described a somewhat similar case above, in which both the median incisors were geminous. An interesting case of a somewhat similar nature is described by Dr. Kirk. It occurred in the lower jaw in connection with the milk dentition. The right lateral was normal, the right median was twice its customary breadth and divided longitudinally into two halves by a deep groove, extending from the middle of the cutting edge of the crown to the lower end of what remained of the fang, giving the appearance of two teeth fused together. The root of this tooth was unfortunately so absorbed that its condition, whether single or double originally, could not be made out. The left median was normal, and its fang was about half absorbed. The left lateral was twice its ordinary breadth and longitudinally divided by a groove like the right median, and the root was unabsorbed and distinctly bifid for at least a third of its length.

The permanent teeth presented on their appearance no abnormality.

Cases in which five normally-formed incisors have been observed are fairly numerous, and seven such are included in our series. Supernumerary teeth are even more numerous both in our own experience and in that of other observers. We have already described five cases in which there were two, and fifteen in which there was one. One case shows an unsuccessful attempt at reversion to the full dentition; here a small incisiform tooth is placed behind the right lateral and in series with it, and a blunt supernumerary tooth posterior to the left median. Mr. J. S. Amoore has afforded us information of an interesting case in which a supplemental tooth in the temporary series had a permanent successor. This case throws much light upon the question of the derivation of such teeth. Viewed from the light of atavism, these cases afford fresh confirmation of the theory that man's incisor dentition was originally In $\frac{3-3}{3-3}$. It is seldom that he attains to it in the upper jaw, still less frequently in the lower, never, so far as we are aware, in both simultaneously. But, on the other hand, in quite sufficiently numerous cases he regains one of his lost teeth, either well or ill-formed, or both in an imperfectly-formed condition; and, finally, this gain may occur in both dentitions.

II. *Man's lost incisor is the lateral or In3.*

We approach this portion of our subject with much greater diffidence, since the position which we maintain differs from that which has the weight of Professor Turner's name attached to it, and from the opinion which Baume has advanced. We shall first consider the view put forward both by Baume and by Edwards, as that which seems to us least tenable. The two chief reasons advanced by Baume are, firstly, that a diastema exists sometimes in apes and in man between the central incisors, and secondly, that supernumerary teeth are found either between the median incisors or immediately behind them. With respect to the first argument we are bound to say that our own experience in no way confirms Baume and Edwards' statements respecting the frequency of occurrence of a separation between the median incisors. For some considerable time we have made observations upon this point, and are inclined to believe that any noteworthy separation at the point indicated is, at least in this district, uncommon. And it must be remarked that, as dental abnormalities are numerous amongst the class of patients attending at the Dental Hospital in this town, we should certainly have expected to find a fairly large number of instances of the separation, were it the marked feature which it has been claimed to be. In connection with the second argument, we think that any one who has examined such a series of models as are at our command will be struck at once by the fact, that whatever teeth are added or suppressed, the two medians typical in shape or invariably, or almost absolutely so, present.

Thus in the case of six incisors, if the question be doubtful whether $In_2$ or $In_3$ be the intruder, at least it is obvious that $In_1$ is the same in shape as the central in any normal series.

It is quite true that supernumerary teeth exist frequently in the middle line, but very rarely to the exclusion of the median. Moreover, we have specimens which prove beyond a doubt that the present lateral incisors may take up their position behind the median, which at least goes to prove that a tooth in this position need not necessarily be a suppressed median. We have specimens showing this occurrence in both upper and lower jaw, and affecting one or both laterals.

Finally, there are two other arguments based upon the dentition of Homalodontotherium and the undoubted suppression at present occurring of the lateral incisor, both of which will be dealt with at greater length in considering Professor Turner's theory, and both of which apply with even greater force to the refutation of that of Baume and Edwards.

Turner's theory is based upon facts learnt from a study of a number of cases of alveolar fissure. It is needless for us to repeat his remarks, which we have quoted at an earlier period. Their weight must be felt by all who have studied the subject, and we can offer at present no explanation of the apparent contradiction which the facts he cites give to our theory. We would, however, venture to urge the following points in support of the view which we have taken that the lost incisor is $In_3$ .

1. The theory that man possessed six incisors is amongst other things based upon the dentition of Homalodontotherium. The dentition of this animal is, according to Tomes, "chiefly instructive in that the teeth in close juxtaposition one with another present an exceedingly perfect gradation of form from the front to the back of the mouth, no tooth differing markedly from its neighbor, though the difference between, say, the first incisor and first molar is exceedingly great. In Professor Flower's words, 'It is only by the analogy of other forms that they can be separated into the groups convenient for descriptive purposes, designated as incisors, canines, premolars, and molars.' In viewing the gradational characters which do exist between the various human teeth, it must not be forgotten that some links in the chain have dropped out and are absent. Mention has already been made of the full typical number of mammalian teeth being 44, that is:—

$$In\frac{3-3}{3-3},\ C\frac{1-1}{1-1},\ PM\frac{4-4}{4-4},\ M\frac{3-3}{3-3}=44.$$

The human subject does not possess the third incisors nor the first two premolars, so that a somewhat abrupt change of form in passing from the incisors to canines, and from the latter to the bicuspids is no more than might be anticipated."

It will be seen from this quotation that Tomes puts forward the theory which we support upon the evidence of Homalodontotherium. If we suppose either $In_1$ or $In_2$ to be the missing tooth, the gradation theory falls to the ground unless indeed we assume that *pari passu* with the suppression of one or other of these teeth, the remaining become modified in form, so that the gradual alternation in shape to the canine is lost, an assumption which is not supported by any facts of which we are aware.

2. It has long been held, and the facts which have been brought forward, together with those which we have yet to deal with, appear amply to warrant the belief that the lateral incisor $In_2$ is at present being gradually suppressed. This being so, it seems *prima facie* much more likely that the tooth which has already been lost is that which was next behind it in the original dental series.

3. In all those cases in which there are six or five incisor teeth in series, the additional tooth is, in our opinion, indisputably placed behind the lateral incisor, and is, in fact, as Dr. Albrecht and Professor Turner have styled it a "precanine." This feature is well shown in one of our specimens, where a small incisiform tooth is placed behind the true lateral and in series with it. Another again affords an admirable example of a similar state of affairs. On the right hand there are present $In_1$ corresponding to $In_1$ on the left, $In_2$ corresponding in like manner to its fellow of the opposite side, and behind it, and in a series with it, is an $In_3$ which forms a good instance of a tooth tending towards the shape of the canine, and bridging over the interval in shape normally observed between this tooth and that of the region in front. We believe that an examination of specimens of superfluous incisor teeth placed in proper series will go entirely to prove that the suppressed incisor is the third, and the argument thus supplied we consider far more conclusive than either of the others which we have advanced, valuable as they are, as corroborative evidence.

III. *This loss is consequent upon the contraction of the anterior part of the jaw.*

The fact that the jaws and alveolar arches of higher races are less well formed than those of lower is sufficiently recognized. On this point Oakley Coles writes as follows:—" It seems to have been proved fairly conclusively that irregularities and malformations of the upper jaw and palate are met with much more frequently amongst highly civilized races than amongst those who have lived, or are still living under the semi-barbarous conditions. And it would further appear that the same irregularities are of more frequent occurence amongst the working classes. The two hundred ancient skulls examined by Messrs. Cartwright and Coleman in the crypt of Hythe Church, presented without exception perfect maxillæ and extremely well-developed alveolar arches; while the more extensive researches of Mr. J. R. Mummery, extending to upwards of three thousand skulls of ancient and modern uncivilized races, lead to the conclusion that the perfect type of both dental and maxillary arches has been uniformly maintained amongst nations of simple habits and lives. Again, Dr. Nichols of New York, who has examined the mouths of thousands of Indians and Chinese, affirms that the jaws of both races are universally well formed and amply developed."

Darwin and Herbert Spencer have both noticed the decrease in size of the jaws in civilized races as compared with uncivilized, and in the upper classes of the former as compared with the lower. The latter states that the maxillæ of Australians, Negroes, and ancient British are absolutely larger than those of the modern English, and, when considered in connection with their smaller skeletons, the difference is of course very much more marked. We are inclined to believe, though we have not yet had an opportunity of working out the subject as fully as we could wish, for lack of material, that this decrease in size is exhibited in perhaps the most marked manner in the incisive region. Topinard states that the elliptical form of jaw is that which is most commonly met with among inferior races, the parabolic being that which more peculiarly belongs to the superior. From an examination of these two types of jaw the former certainly appears to afford a much more roomy incisive region.

A comparison between the various casts in our possession of English teeth and those casts and skulls of uncivilized races which we have been able to examine, leads us to the conclusion that the incisive region in the latter is much less contracted than that of the former.

In a paper "On the so-called Serpent Teeth" Callender puts forward the theory that certain abnormalities of the incisive region may depend upon arrested or stunted growth of the incisive process of the superior maxilla. The same theory will help to account for the contraction of the incisive region leading to the suppression of teeth. If the incisive process be stunted in its growth then the proper development of the inter-maxillary bone is prevented and the incisors are thrown inwards or suppressed.

When it is considered that the incisor teeth are those used for cutting or dividing food, and when it is remembered how much their labors have been lessened amongst civilised races by the careful cooking of food, there will be no difficulty in assigning a reason for the gradual disappearance of these particular members of the dental series.

IV. *Suppression of the two present lateral incisors is now taking place.*

Cope has ventured to predict that in future the lower races of men will retain the dentition of the present day, whilst those intellectually higher will possess a diminished formula of

$$\text{In}\frac{1-1}{1-1},\ \text{C}\frac{1-1}{1-1},\ \text{PM}\frac{2-2}{2-2},\ \text{M}\frac{3-3}{3-3}$$

or

$$\text{In}\frac{1-1}{1-1},\ \text{C}\frac{1-1}{1-1},\ \text{PM}\frac{2-2}{2-2},\ \text{M}\frac{2-2}{2-2}$$

and the view which he puts forward as to the disappearance of the incisors is shared by other authorities on odontological subjects, whose opinions have been quoted in an earlier part of this communication.

We think that our Series, IV. to VIII. inclusive, afford good examples of this process of suppression in its various stages. Series IV. contains cases in which there are at present three well-formed incisors and one of lesser development, a conical or supernumerary tooth. Series V. shows a still further advance in the substitution of a supernumerary tooth for one of the three still left of the normal shape in the former category. In Series VI. one tooth has actually disappeared. In Series VII., besides the suppression of one tooth, we have malformation or imperfection of a second, whilst in Series VIII. we finally reach the complete condition of suppression of both lateral incisors. We quote the ascertained ages as far as possible of the cases in this series, in order to show that these are not cases in which from immaturity the missing teeth might yet present themselves. In one case, three incisors, one being conical, existed in the superior maxilla, two only in the lower, those present and perfect being the central. This is interesting as being the closest approach to the reduced dentition predicted by Cope with which we are acquainted. It will be noticed that, of twenty-five cases included in this series, twenty-three are instances of suppression of greater or less degree of the lateral incisors, two only of the median.

Amongst these cases of suppression are three which form an intesesting family group. The parents of these three children belong to the upper classes, were not related to one another, and present themselves no dental abnormalities. The eldest child, a girl aged 22, has neither lateral incisor in the upper jaw; the second, also a girl aged 20, wants the right lateral; whilst the third, a boy aged 17, wants, like the eldest, both the permanent laterals, but the deciduous right lateral is still present.

We think that, so far as the evidence from dental abnormalities goes, the case for the present suppression of the lateral incisors may be considered to be fairly complete.

V. *Conical Teeth a Reversion to tle Primitive Type.*—That the simplest form of tooth is the conical is an obvious fact from Comparative Anatomy. It is interesting to observe, then, that in most of the cases in which an attempt at a reversion to the full dentition is being made by nature, and others in which a portion of the present dentition is being suppressed, the abnormal tooth, being apparently unable to attain to its full development, remains of the same shape in which in lower animals is normal.

It is instructive to find that this reversion to the conical shape may be found in supernumerary teeth in other animals. We have a skull of a *Midas rosalia*, in which in the upper jaw there is a supernumerary tooth which we consider to be a premolar. This tooth has the same conical form as the supernumeraries in Man, and differs thus very markedly from the shape of its fellows of the same series.—*London Dental Record.*

